# Prevalence of Post‐Operative Complications in Autotransplanted Teeth: A Long‐Term Retrospective Cohort

**DOI:** 10.1111/edt.70038

**Published:** 2025-12-02

**Authors:** Juraj Marton, Radovan Žižka, Linda Kučerová, Přemysl Krejčí, Martin Starosta, Zdeněk Pokorný

**Affiliations:** ^1^ Department of Dentistry and Oral Science, Faculty of Medicine and Dentistry Palacký University Olomouc Olomouc Czech Republic; ^2^ Department of Dentistry, Faculty of Medicine University of Ostrava Ostrava Czech Republic

**Keywords:** autologous transplantation, post‐operative complications, retrospective studies, survival analysis, time factors, tooth resorption

## Abstract

**Background/Aims:**

To evaluate the prevalence and timing of post‐operative complications following tooth autotransplantation, identify factors associated with earlier diagnosis, and report long‐term survival and success rates.

**Materials and Methods:**

A single‐centre retrospective cohort study was conducted at a Czech university dental clinic (2003–2024); no external funding. Donor teeth were grouped as premolars, molars and anterior (canines, incisors, one premaxillary supernumerary; predominantly single‐rooted, single‐canal). Root development was classified according to Moorrees: early (1–3), optimal (4–5), late (6–7). Survival/success were estimated with Kaplan–Meier; timing of first diagnosis with event‐only cumulative incidence (1–Kaplan–Meier); and factors associated with earlier occurrence with Cox proportional hazards regression.

**Results:**

The study included 134 teeth in 111 patients (74 premolars, 48 molars, 12 anterior). Half of the complications were diagnosed within 18 months (median time to first diagnosis: 11.7 months inflammatory resorption, 19.6 months apical pathology, 18.0 months cervical resorption, 21.4 months replacement resorption). Five‐year survival: premolars 95.4%; molars 86.6%; anterior 75.8%. Five‐year success: 75.3%, 76.8% and 66.0%, respectively. Age ≥ 30 years was associated with earlier tooth loss and apical pathology; male gender with earlier inflammatory resorption and loss of success. Anterior teeth and restorative indications (e.g., caries, previous root canal treatment failure) were associated with earlier apical pathology. A single preoperative dose of amoxicillin 1 g ~1 h before surgery or a 7‐day post‐operative amoxicillin course (500–1000 mg) was associated with delayed diagnosis of infection‐related complications. Some treated complications recurred or transformed into another type, requiring additional intervention.

**Conclusions:**

Early detection enables successful management of most complications. As half occurred within 18 months—the critical window—follow‐up should include reviews at 1 week, 1 month, 3 months and every 3 months until 2 years post‐transplantation, with continued long‐term monitoring for recurrences and late complications.

## 1 Introduction

Tooth loss poses a significant clinical challenge across all age groups, particularly in younger patients, where dental implants are often contraindicated due to ongoing skeletal development. In such situations, tooth autotransplantation—a multidisciplinary treatment modality involving the surgical relocation of a tooth within the same individual—serves as a valuable treatment alternative. This approach preserves natural proprioception, allows for ongoing root development and is generally more cost‐effective than implant‐supported restorations. Nonetheless, the procedure carries specific risks, notably complications related to pulp healing, root integrity and periodontal ligament (PDL) function. These complications may manifest as inflammatory root resorption, apical pathology, external cervical resorption or ankylosis, each of which can compromise long‐term outcomes.

International literature reports encouraging survival and success rates for autotransplanted teeth, often comparable to dental implants, even with extended follow‐up periods and large patient cohorts [1, 2]. Despite a longstanding clinical tradition of tooth autotransplantation in the Czech Republic [3], published data from this region remain limited, typically originating from small sample sizes with short‐term follow‐up and rarely exceeding 60 transplanted teeth [4]. Notably, since the 1960s, no large‐scale, long‐term cohort study on tooth autotransplantation has been published from the Czech Republic.

Although overall survival rates and complication prevalence have been well documented internationally, fewer studies have focused on the timing of complications and the diagnostic windows in which they are detected [5]. Early diagnosis is essential, as most complications can be effectively managed if identified promptly. For example, root canal treatment (RCT) may arrest inflammatory root resorption [6, 7]; removal of resorptive tissue followed by restorative treatment can control external cervical resorption [8]; and, in selected cases, attempts have been made to manage early ankylosis by luxation and orthodontic movement [9]. According to recognised success criteria, teeth with appropriately treated complications may still be considered successful at long‐term follow‐up [10, 11]. Similarly, while factors such as patient age [12], stage of root development [13] and antibiotic use [14] are acknowledged to influence the incidence of complications, their impact on the timing of these events remains insufficiently studied.

This single‐centre retrospective cohort study aimed to assess the prevalence and timing of post‐operative complications following autotransplantation of premolars, molars and anterior teeth (a heterogeneous group of predominantly single‐rooted, single‐canal donors, including canines, incisors and one premaxillary supernumerary tooth) over a long‐term follow‐up period. Additionally, the study investigated potential associations between the timing of complication occurrence and selected patient‐, treatment‐ and tooth‐related factors, and reported long‐term survival outcomes.

## 2 Materials and Methods

### 2.1 Study Design, Ethics and Sample

This retrospective observational study was conducted at the Department of Dentistry and Oral Science, Faculty of Medicine and Dentistry, Palacký University Olomouc, Czech Republic. The dataset included patients who underwent tooth autotransplantation between January 2003 and September 2024. The protocol was approved by the Ethics Committee of the University Hospital and Faculty of Medicine and Dentistry of Palacký University Olomouc (Reference No. 105/25) and adhered to the ethical standards of the 2013 Declaration of Helsinki. Owing to the retrospective design and use of anonymised data, the requirement for individual patient consent was waived by the committee. The study complies with the STROBE 2013 checklist and is reported in accordance with the PROBE 2023 guidelines. Data collection was finalised on 15 September 2024.

Patient records were identified using the search function of the PC Dent software, querying the keyword ‘autotransplant’ across medical notes from all departments. The study population included patients referred for autotransplantation from other departments within the university dental clinic (e.g., orthodontics), as well as from external practices. In total, 256 cases were initially identified. Manual verification excluded unrelated procedures (e.g., bone autografts or unclear documentation; 94 cases) and cases with < 1 month of follow‐up without tooth loss (15 cases). Radiographic data were retrieved from two imaging systems (Romexis and Digora). The final dataset comprised 134 autotransplanted teeth in 111 patients. Most procedures (*n* = 100) were performed at the Department of Periodontology, with the remainder (*n* = 34) at the Department of Paediatric Dentistry. In total, nine surgeons carried out transplantations during the study period.

### 2.2 Presurgical Phase

Preoperative consultation comprised a medical history questionnaire, clinical examination and radiological assessment. Intraoral examination included evaluation of oral hygiene, periodontal assessment and inspection for caries. Clinicians measured the dimensions of both the donor tooth and the recipient site. Panoramic and intraoral radiographs were routinely used to assess the donor tooth morphology, position and stage of root development; in selected cases, cone beam computed tomography (CBCT) was employed for more complex evaluation.

More than half of the cases were referred from orthodontic practices, where space opening in the recipient site had already been completed. In some instances, the surgeon requested further orthodontic widening of the recipient space to exceed the donor tooth's dimensions and ensure passive insertion. Occasionally, space was also created around the donor tooth to facilitate atraumatic extraction. In orthodontically treated cases after 2019, preloading was commonly applied 2 weeks prior to surgery to enhance PDL responsiveness [15, 16]. Surgery was postponed in selected cases to permit further root development or to manage local contraindications such as caries or periodontal disease, which were addressed prior to the procedure. Patients received oral hygiene instruction and professional cleaning before surgery to reduce bacterial load and optimise healing.

### 2.3 Surgical Procedure

Autotransplantation was performed by three approaches: immediate, involving placement into a fresh extraction socket at the same visit; semi‐immediate (delayed), in which the recipient tooth was extracted first and the transplant was placed 2–5 weeks later [17, 18], while the native socket contour was still largely preserved and before substantial osseous remodelling—this permitted resolution of local inflammation and soft‐tissue thickening; and conventional, with transplantation into a fully healed site after creation of a surgically prepared recipient bed (neo‐socket).

When recipient socket preparation was required, it was performed using implant drills and carbide burs under saline irrigation, following full‐thickness flap elevation. Donor teeth were carefully extracted, predominantly using forceps, with no or minimal use of elevators and no subgingival intervention.

If adjustments to the recipient site were necessary, the donor tooth was temporarily stored in sterile saline‐soaked gauze or in the original extraction socket. In a subset of cases, 3D‐printed tooth replicas (CARP models) were used by one participating surgeon to guide socket shaping and improve accuracy.

Following flap repositioning, initial stabilisation was achieved using two cross‐mattress sutures covered by flowable composite. In some cases, stabilisation to an orthodontic appliance (without active forces) or to adjacent teeth using orthodontic wire and composite was employed. One surgeon consistently used square orthodontic wire and composite for primary stabilisation. When ideal infraocclusion was not initially achievable, temporary composite bite stops were bonded to neighbouring teeth to disclude the transplant and act as a protective occlusal pivot during early healing. Thereafter, occlusion was adjusted and/or orthodontic movement initiated as indicated, and the temporary build‐ups were removed.

Antibiotics were routinely administered, most commonly as a single prophylactic dose of 1 g amoxicillin approximately 1 h prior to surgery, or as a post‐operative regimen for 7 days starting on the day of surgery (e.g., amoxicillin 500–1000 mg or clindamycin 300–600 mg in penicillin‐allergic patients).

### 2.4 Post‐Operative Follow‐Up

Sutures were typically removed after 1 week. If the transplant remained mobile, sutures were either left in situ for a further week or the tooth was stabilised to an orthodontic appliance or to adjacent teeth with wire and composite; patients were then reviewed weekly until stability was achieved (usually up to the 1‐month visit). Thereafter, the routine schedule consisted of visits at 1, 3, 6 and 12 months, and subsequently at six‐monthly intervals. During follow‐up, patients were also reviewed by other specialists (e.g., endodontists, orthodontists) as required. At surgical team visits, clinical and radiographic assessments were conducted, with periapical radiographs obtained to monitor apical healing and detect resorptive changes.

At follow‐up, clinicians routinely assessed oral hygiene, performed periodontal examinations, tested pulp sensibility (cold test, electric pulp test), and evaluated mobility and percussion responses—screening for both pain and ankylosis. Visual inspection of the crown (e.g., pink discolouration) and surrounding gingiva (e.g., inflammation or hyperplasia) was also performed to detect signs of external cervical resorption (ECR). Periodontal indices, including probing depth (PD), gingival margin level (GML), bleeding on probing (BOP) and plaque score, were commonly recorded. However, detailed recordings of PD, GML, BOP and Periotest values were only available in a subset of the more recent cases, due to the clinic's earlier use of hybrid documentation—paper periodontal charts (now discarded) in conjunction with brief electronic records. All clinically significant findings were documented electronically and included in the final analysis.

For teeth with late root development, the endodontic approach differed by Moorrees' stage. In stage 7, RCT was performed before transplantation or within 14 days in the later years of the study (2010–present), with appointments prearranged at the surgical consultation, in accordance with published recommendations [11, 19]: In earlier years (2000–2010), some stage 7 cases underwent RCT later (approximately 2 months post‐operatively) due to scheduling constraints. In stage 6, pulpal sensibility testing and radiographic review were undertaken; RCT was performed if pathology developed or if the tooth remained non‐responsive to sensibility testing at 12 months. Otherwise, evidence of revascularisation led to deferral of RCT [5, 20].

Orthodontic tooth movement was initiated once periodontal stability had been confirmed clinically and radiographically, typically 3–6 months after transplantation. In teeth at early root development stages (Moorrees' stages 1–3), movement was postponed until sufficient root length had been achieved to minimise the risk of accidental extraction. Early movement was undertaken in selected cases showing unusually rapid firming with minimal mobility, with the aim of maintaining PDL activity and reducing the risk of ankylosis, while applying only light, controlled forces [21].

### 2.5 Data Collection

Clinical records and radiographic images were reviewed independently by two investigators, with disagreements resolved through discussion and consensus. For patients referred from other departments within the dental clinic, medical records from the referring department were also examined to capture additional events or relevant history.

Collected variables included:

#### 2.5.1 Demographics

Age at surgery; gender.

#### 2.5.2 Procedural Details

Date of surgery and last follow‐up; donor tooth type; recipient site; indication for transplantation (agenesis or trauma to anterior teeth; impaction; restorative indications, e.g., caries or failure of previous RCT); timing of surgery relative to extraction of the recipient tooth (immediate, semi‐immediate, conventional); surgeon's experience level (≥ 20 or < 20 procedures performed); root development stage (converted to Moorrees' classification [22]); splinting method (sutures covered with composite, orthodontic wire with composite, conversion from suture to wire or orthodontic appliance) and duration; antibiotic regimen (agent, single preoperative dose or 7‐day post‐operative course); perioperative or post‐operative donor tooth adjustments (occlusal grinding, root resection, crown restoration); use of 3D‐printed replicas during socket preparation; use of composite bite stops; timing of RCT in mature teeth (before transplantation or ≤ 14 days; > 14 days or symptom‐triggered).

#### 2.5.3 Clinical Parameters

PD, GML, BOP, clinical attachment loss (CAL), mobility (Miller mobility index [23], Periotest value), percussion sound (for ankylosis detection), pulp sensibility testing (cold test or electric pulp test), gingival condition and crown discolouration.

#### 2.5.4 Radiographic Parameters

Root development; PDL space and continuity of lamina dura; pulp canal obliteration (an indicator of revascularisation); periapical radiolucency—a sign of apical pathology (AP); and resorptive changes—internal inflammatory resorption (IIR), defined as a progressive well‐circumscribed radiolucent enlargement of the pulp canal or chamber [24, 25]; external inflammatory resorption (EIR), defined as an extensive resorptive area with associated loss of lamina dura (radiolucency) [7, 26]; external cervical resorption (ECR), defined as a cervical radiolucency located supracrestally at or near the cemento‐enamel junction (sometimes with a visible point of entry) and displaying a mottled or mixed radiolucent‐radiopaque pattern [27, 28]; and replacement resorption (RR), defined as loss of lamina dura with replacement of dental tissues by bone [7].

**FIGURE 1:**
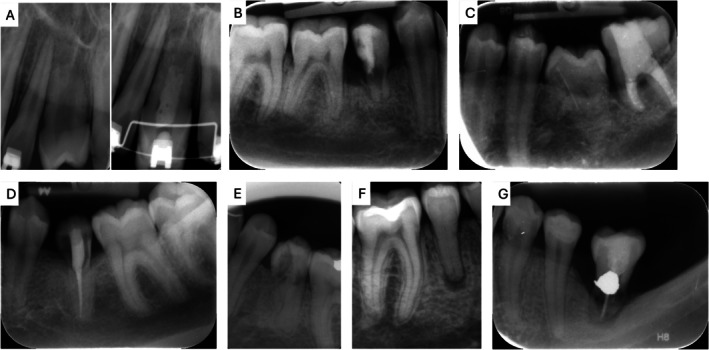
Representative radiographic examples of the main post‐operative complications. (A) External inflammatory resorption (EIR): initial diagnosis (left), first application of calcium hydroxide (right); (B) Internal inflammatory resorption (IIR); (C) Replacement resorption (RR); (D) External cervical resorption (ECR) with vascular soft‐tissue ingrowth; (E) ECR with osseous tissue ingrowth; (F) Apical pathology (AP); (G) Periodontal attachment loss (AL) with concurrent resorption.

All suspected lesions were evaluated using both clinical and radiographic records from follow‐up examinations, including documentation from other departments where applicable. In diagnostically challenging cases (e.g., differentiation between resorptive complications), where limitations of intraoral radiographs hindered definitive assessment, CBCT imaging—often performed by the treating practitioner for diagnostic and treatment purposes—was additionally reviewed to support diagnosis. Representative radiographic examples of EIR, IIR, AP, ECR and RR are shown in Figure 1.

#### 2.5.5 Outcomes

Tooth loss was defined as extraction of the transplant during the observation period; the reason for tooth loss was recorded (e.g., progressive resorption, persistent AP with symptoms despite endodontic treatment or periodontal attachment loss). Periodontal attachment loss (AL) encompassed both failure of periodontal healing in the early phase and progressive loss during follow‐up, evidenced by an increase in CAL together with radiographic bone loss [29]. Complications included AP, EIR and IIR, ECR, RR (i.e., ankylosis). For each event, the date of first diagnosis and the time from surgery to diagnosis were documented.

Success was defined according to Tsukiboshi [11] as a transplanted tooth functional at the last follow‐up, with normal mobility, absence of discomfort, no signs of inflammation or periodontal pocket formation, and radiographic evidence of a normal PDL space and intact lamina dura, without signs of progressive root resorption, ankylosis or AP. Successfully treated and stabilised resorptions without progression were considered compatible with success [10]. In contrast, loss of success was recorded if a complication remained untreated at the last recall—even if potentially treatable but unresolved at that time (‘worst‐case scenario’)—or if it progressed despite treatment; the date of loss of success was defined as the date the complication was first diagnosed. Survival was defined as the presence of the transplanted tooth irrespective of clinical status.

Dual independent review with consensus resolution was used to enhance the reliability of assessments. Periapical radiographs were used for monitoring, acknowledging their lower sensitivity compared to CBCT. When available, CBCT scans were reviewed to improve diagnostic certainty. In cases where a suspected complication at the last recorded appointment could not be confirmed radiographically, the diagnosis was verified with the referring dentist. Applying a strict ‘worst‐case scenario’ approach to defining success may have resulted in underestimation of the true success rate.

### 2.6 Statistical Analysis

Statistical analysis was conducted using IBM SPSS Statistics (Version 31; IBM Corp., Armonk, NY, USA). The primary unit of analysis was the transplanted tooth. To account for potential clustering, key analyses were repeated in a patient‐level sensitivity set that included only the first transplant per patient.

Kaplan–Meier (KM) methods were used for survival (event: extraction) and success (event: loss of success according to Tsukiboshi's criteria). KM analyses were stratified by donor tooth type (anterior, premolar, molar). Additional KM curves stratified by root development (Moorrees' groups) and by RCT timing (before transplantation or ≤ 14 days vs. > 14 days or symptom‐triggered) are presented in the Supporting Information. Log–rank tests were reported in the figure footnotes. Censoring was defined as the last recorded follow‐up.

Complications diagnosed during the observation period and reasons for tooth loss were summarised descriptively in tables as counts (*n*) and percentages, stratified by donor tooth type (anterior, premolar, molar). For each complication, the status at last review was recorded (successfully treated/stable; under treatment or progressing; resulted in tooth loss), and evolution to another complication type or subsequent failure for a different reason was indicated. Corresponding stratifications by root development (Moorrees' groups) and by RCT timing (late root development stages) are presented in the Supporting Information.

Time to first diagnosis was described using event‐only cumulative incidence curves (1–KM) to characterise the distribution of diagnosis times without confounding by different event rates across groups. Median (IQR) times were reported among event cases. Standard full‐cohort cumulative incidence curves are provided in the Supporting Information, stratified by donor tooth type, root development and RCT timing.

Cox proportional hazards regression was used to evaluate associations between patient‐, tooth‐ and treatment‐related factors and time to first diagnosis of complications. Results were expressed as hazard ratios (HR) with 95% confidence intervals, where HR > 1 indicates earlier occurrence (shorter time to event).

Patient‐related factors included: age (< 18, 18–29 and ≥ 30 years) and gender. The < 18 years cut‐off reflects the completion of root development for many potential donor teeth (e.g., third molars) by late adolescence [22]. The ≥ 30 years threshold was selected because alveolar bone becomes denser and less elastic with age, particularly after the third decade, which may increase extraction difficulty and the risk of complications [30, 31, 32].

Tooth‐related factors included: root maturity, root development stage (Moorrees' stages 1–3 = early, stages 4–5 = optimal, stages 6–7 = late) and donor tooth type (anterior—including incisors, canines and a premaxillary supernumerary tooth; premolars; molars). Anterior teeth were grouped together owing to the small sample size and shared characteristics—anatomically, they were predominantly single‐rooted with a single canal; clinically, they were often impacted and used for similar indications.

Treatment‐related factors included: indication (agenesis or trauma to anterior teeth; impaction; restorative indications, e.g., caries or failure of previous RCT), surgeon's experience (≥ 20 vs. < 20 cases), timing of surgery relative to extraction (immediate, semi‐immediate, conventional), timing of RCT (before transplantation or ≤ 14 days [11, 19] vs. > 14 days or symptom‐triggered [5, 20]), antibiotic regimen (none, single preoperative dose of amoxicillin 1 g, or 7‐day post‐operative course of amoxicillin 500–1000 mg or clindamycin 300–600 mg in penicillin‐allergic patients), splinting duration (≤ 4 weeks vs. > 4 weeks, in accordance with established recommendations for autotransplantation [11]), use of composite bite stops during early healing, post‐operative orthodontic loading (early < 3 months vs. conventional ≥ 3 months), and donor tooth adjustments (e.g., occlusal grinding, root resection, crown restoration).

Variables with sparse data and unstable estimates (very wide confidence intervals) were not interpreted individually, and their results were summarised collectively in a bottom section of the table.

Quantitative variables were categorised as described above, and categorical variables were coded as binary or multinomial, as appropriate. Missing data were uncommon and handled by complete‐case analysis. For orthodontic loading, cases without verifiable timing were excluded from regression analyses; cases with ‘unknown’ timing were reported descriptively in the materials presentation table with counts. Where possible, additional information was obtained from referring clinicians or patients. Censoring was applied at the date of last follow‐up if no event had occurred. No imputation methods or bias correction procedures were applied.

As an additional sensitivity analysis addressing heterogeneity in the anterior donor tooth group, KM and Cox models were repeated with canines only; results are provided in the Supporting Information.

## 3 Results

A total of 134 teeth in 111 patients were analysed (tooth‐level gender distribution: 73 female, 61 male; mean age 19.9 years, range 9–63). The mean observation period was 38 months (range: 0–167). Donor distribution comprised 74 premolars, 48 molars and 12 anterior teeth (7 canines, 4 incisors, 1 premaxillary supernumerary tooth). Root development at surgery comprised 11 early (1–3), 71 optimal (4–5) and 52 late (6–7) stages. Full characteristics, including RCT timing, indication, recipient‐site timing and orthodontic loading, are presented in Table 1.

**TABLE 1:** Total number of transplanted teeth from 2003 to 2024, stratified by donor tooth type and Moorrees' stage.

Donor tooth type	Root development (Moorrees) and treatment protocol	*N*	Gender (*n* (%), ♀/♂)	Age (years, mean [range])	Indication (*n*)			Recipient‐site timing^b^ (*n*)			Orthodontic loading^c^ (*n*; documented cases only; early ≤ 3 months; conventional > 3 months; none)	Observation period^d^ (months, mean [range])
					Agenesis/trauma	Impaction	Restorative^a^	Immediate	Semi‐immediate	Conventional		
Anterior^e^ (*n* = 12) (incisors, canines, supernumerary)	Optimal (4–5)	4	♀2/♂2	12 [9–14]		3	1	2		2	1; 2; 1	19 [3–33]
	Late (6–7)—RCT > 14 days or symptom‐triggered	6	♀4/♂2	26.7 [12–55]	2	3	1	4		2	3; 1; 1	32 [5–79]
	Late (7)—RCT before or ≤ 14 days	2	♀0/♂2	13 [12–14]	1	1		2			1; 1; 0	66 [51–81]
Premolars (*n* = 74)	Early (1–3)	4	♀2/♂2	12 [10–13]	3	1		3		1	1; 1; 0	77 [29–141]
	Optimal (4–5)	54	♀29/♂25	12.8 [10–19]^f^	48	1	5	38	1	15	23; 16; 1	44 [1–128]
	Late (6–7)—RCT > 14 days or symptom‐triggered	14	♀8/♂6	18 [12–31]	10	1	3	9		5	8; 1; 1	42 [5–96]
	Late (7)—RCT before or ≤ 14 days	2	♀1/♂1	15 [14–16]	2			2			0; 1; 0	38 [9–68]
Molars (*n* = 48)	Early (1–3)	7	♀4/♂3	16.6 [15–19]	1		6	2		5	1; 0; 5	32 [1–89]
	Optimal (4–5)	13	♀8/♂5	16.6 [11–22]	3		10	7	1	5	3; 1; 8	23 [2–90]
	Late (6–7)—RCT > 14 days or symptom‐triggered	24	♀13/♂11	38.1 [18–63]	2		22	10	4	10	3; 0; 21	36 [0–167]
	Late (7)—RCT before or ≤ 14 days	4	♀2/♂2	43.3 [35–55]			4	1		3	0; 0; 4	6 [3–9]
Total	All stages	134 (100%)	♀73/♂61	19.9 [9–63]	72 (53.7%)	10 (7.5%)	52 (38.8%)	80 (59.7%)	6 (4.4%)	48 (35.8%)	44 (32.8%); 24 (17.9%); 42 (31.3%)	38 [0–167]

^a^ Restorative indications: caries, failure of previous root canal treatment, etc.

^b^ Recipient timing: immediate = fresh extraction socket at the same visit; semi‐immediate = 2–5 weeks after extraction, while native socket contour persists; conventional = surgically crafted neo‐socket.

^c^ Orthodontic loading: counts reported only for cases with documented timing.

^d^ Observation period 0 months: tooth loss within the first month after transplantation.

^e^ Anterior teeth: heterogeneous group of predominantly single‐rooted, single‐canal donors (often impacted): 7 canines, 4 incisors, 1 premaxillary supernumerary.

^f^ Patient aged 19 years with Moorrees stage 5 premolar had delayed dental development and multiple tooth agenesis. Maturity was determined by Moorrees stage rather than chronological age.

**FIGURE 2:**
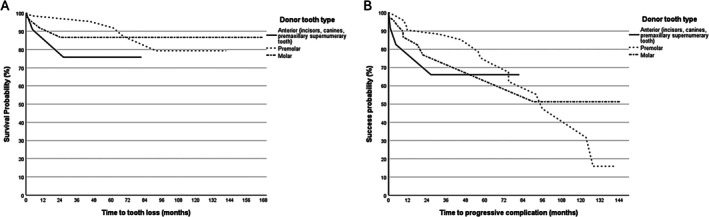
Kaplan–Meier survival and success curves stratified by donor tooth type. The anterior group comprises a heterogeneous set of predominantly single‐rooted, single‐canal donors, often impacted and with similar clinical indications. No between‐group differences were observed (log–rank: survival *p* = 0.260; success *p* = 0.766). Numbers at risk in 12‐month intervals are provided in this figure. Stratification by Moorrees' stage and timing of root canal treatment is presented in the Supporting Information.

**FIGURE 3:**
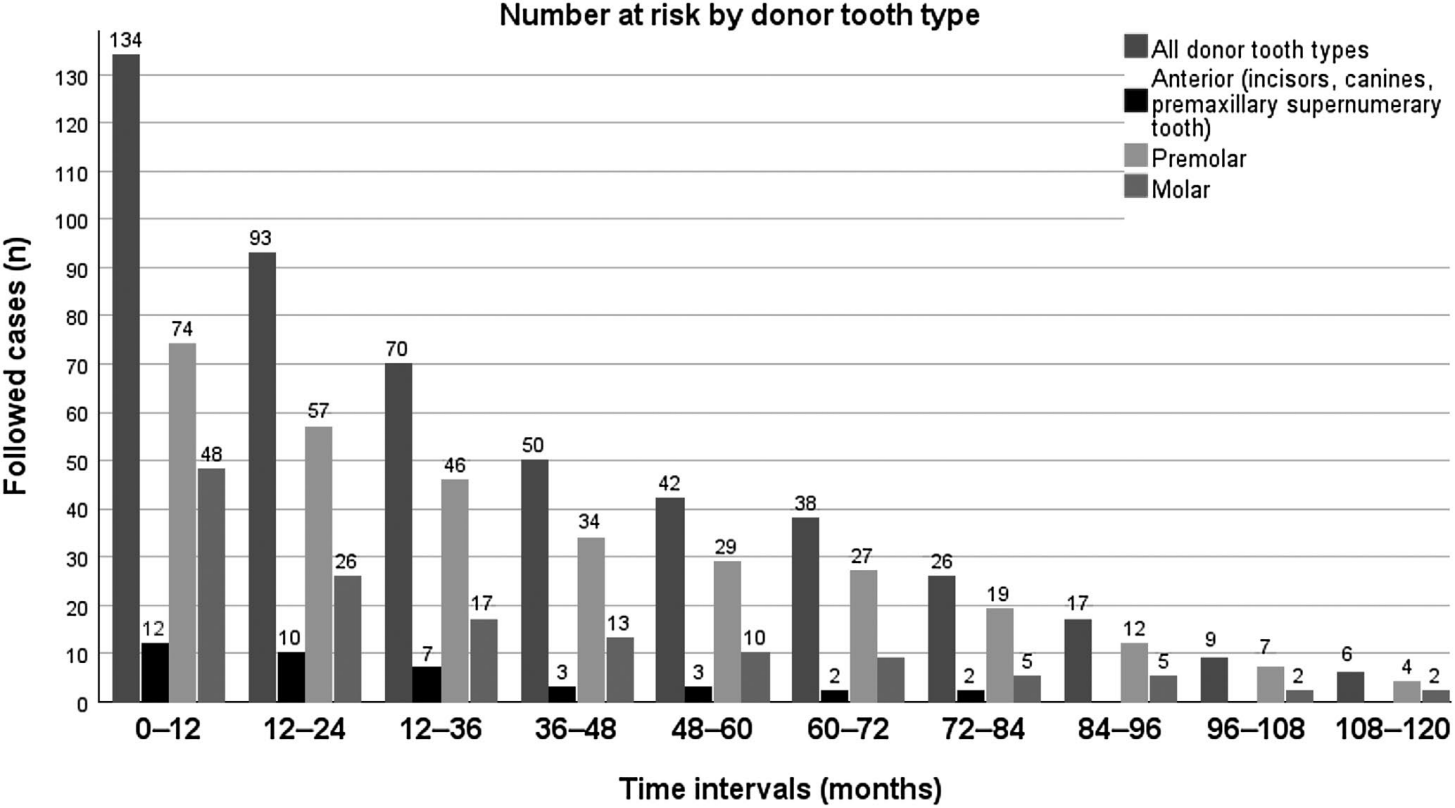
Numbers at risk by donor tooth type (12‐month intervals). Counts correspond to the cohorts shown in Figure 2; see Figure 2 for the definition of the anterior category. Stratification by Moorrees' stage and root canal treatment timing is presented in the Supporting Information.

Survival and success rates by donor tooth type are shown in Figure 2. Five‐year survival rates were 75.8% for anterior teeth, 95.4% for premolars and 86.6% for molars; 5‐year success rates were 66.0% 75.3% and 76.8%, respectively. Between‐group differences were not statistically significant (log–rank *p* = 0.260 for survival; *p* = 0.766 for success). Estimates beyond approximately 7 years should be interpreted with caution owing to the small number at risk (Figure 3). KM curves stratified by root development (Moorrees' groups) and by RCT timing (mature teeth) are provided in the Supporting Information. Corresponding 5‐year survival rates were 100% (early 1–3), 91.9% (optimal 4–5), 87.7% (late 6–7; > 14 days/symptom‐triggered RCT) and 75.0% (late 7; ≤ 14 days), with 5‐year success rates of 59.3%, 77.4%, 67.8% and 58.3%, respectively (Figure S1a,b); the corresponding log–rank tests showed no significant differences (survival *p* = 0.255; success *p* = 0.446). Estimates should be interpreted in light of small subgroup sizes (Figure S1c). A canines‐only sensitivity analysis replacing the anterior group is presented in Figure S2 (5‐year success 100%).

Complications occurred in 44 out of 134 teeth (32.8%), and 12 teeth were extracted during follow‐up. Table 2 presents the distribution and status of complications by donor tooth type; stratifications by Moorrees' stage and RCT timing are provided in Table S1. The most frequent entities were EIR, AP and RR. Resorptive complications were the leading cause of tooth loss (9 out of 12: 5 EIR, 3 ECR, 1 IIR). Three additional extractions were attributed to AL, one concomitant with AP. Multiple concurrent or sequential complications were documented in several cases; seven such teeth were extracted.

**TABLE 2:** Status of autotransplanted teeth at last recall: overview of complications by donor tooth type.

Status	Anterior (*n* = 12)	Premolars (*n* = 74)	Molars (*n* = 48)	All (*n* = 134)	Co‐occurring/subsequent events
**Surviving**	10 (83.3%)	69 (93.2%)	43 (89.6%)	122 (91.0%)	
Without complication	6 (50.0%)	49 (66.2%)	35 (72.9%)	92 (68.7%)	
With complication	4 (33.3%)	20 (27.0%)	8 (16.7%)	32 (23.9%)	
Successfully treated					
EIR		8 (10.8%)	1 (2.1%)	9 (6.7%)	Premolars: 3 subsequently developed RR, including 1 that later failed due to RR
IIR		1 (1.4%)		1 (0.7%)	
AP	2 (16.7%)	4 (5.4%)	2 (4.2%)	8 (5.9%)	Premolars: 2 also had EIR (treated), 1 subsequently developed RR; Molars: 1 later failed due to EIR
RR	1 (8.3%)			1 (0.7%)	
ECR		2 (2.7%)	1 (2.1%)	3 (2.2%)	Premolars: 1 later failed due to combined AP + IIR
Progressing/under treatment					
EIR			2 (4.2%)	2 (1.5%)	
IIR		1 (1.4%)	1 (2.1%)	2 (1.5%)	
AP		2 (2.7%)	2 (4.2%)	4 (3.0%)	Molars: 1 also had EIR, 1 also had IIR
ECR	1 (8.3%)	3 (4.1%)	1 (2.1%)	5 (3.7%)	
RR		7 (9.5%)	2 (4.2%)	9 (6.7%)	Molars: 1 also had ECR
**Tooth loss (extraction)**	2 (16.7%)	5 (6.8%)	5 (10.4%)	12 (9.0%)	
Progressing complications present at extraction					
EIR		1 (1.4%)	3 (6.2%)	4 (3.0%)	
IIR		2 (2.7%)		2 (1.5%)	
AP	1 (8.3%)	1 (1.4%)	1 (4.2%)	3 (2.2%)	Anterior: also had AL; Premolars: also had IIR; Molars: 1 also had EIR
RR		2 (1.4%)	1 (2.1%)	3 (2.2%)	Premolars: 1 also had ECR; Molars: 1 also had ECR
ECR		1 (1.4%)	1 (2.1%)	2 (1.5%)	
AL	2 (16.7%)		1 (2.1%)	3 (2.2%)	
**Teeth with** ≥ **2 complications**	1 (8.3%)	8 (10.8%)	6 (12.5%)	15 (11.2%)	

*Note:* Percentages are calculated column‐wise.

Abbreviations: AL, periodontal attachment loss; AP, apical pathology; ECR, external cervical resorption; EIR, external inflammatory resorption; IIR, internal inflammatory resorption; RR, replacement resorption.

Among surviving teeth with complications, 14 were successfully treated and remained free of progression or symptoms at the last recall, fulfilling the criteria for success. AP, IIR and EIR were managed with RCT; ECR was treated by surgical access, removal of resorptive tissue, and restoration with mineral trioxide aggregate (MTA) or glass ionomer cement (GIC). Early RR was treated by surgical luxation with light orthodontic movement; although initial mobility was achieved, most teeth re‐ankylosed during follow‐up, with only one maintaining mobility (short observation period). In some cases, management of one complication was followed by the development of another: a few successfully treated EIR cases later progressed to RR; RR managed by luxation was occasionally followed by ECR at the treated site; and a rare case of successfully treated AP developed EIR several years later. Repeated interventions were sometimes required (e.g., replacement of restoration after ECR management).

**FIGURE 4:**
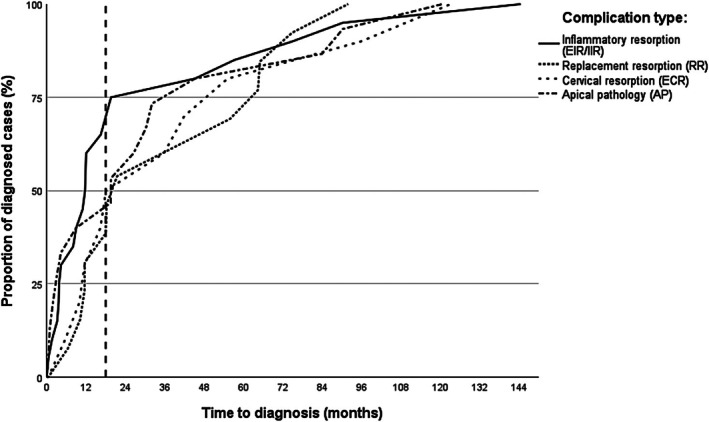
Event‐only cumulative incidence (1–Kaplan–Meier) of post‐operative complications. Separate lines are shown for each complication type; external and internal inflammatory resorption are combined owing to small numbers. The vertical dashed line at 18 months marks the critical interval within which 50% of all complications were diagnosed. Full‐cohort cumulative incidences are presented in the Supporting Information.

Figure 4 (event‐only 1–KM cumulative distributions) illustrates the timing of first diagnosis for inflammatory resorptions (EIR/IIR), RR, ECR and AP in a single display. Median (IQR) time to first diagnosis was 11.7 (3.7–19.6) months for inflammatory resorptions, 21.4 (11.6–64.4) months for RR, 18.0 (11.5–55.2) months for ECR and 19.6 (3.0–45.1) months for AP. A common 18‐month reference line is overlaid; across all complications, 50% were diagnosed within the first 18 months after surgery (median 18.0; IQR 9.0–55.9). Standard full‐cohort cumulative incidences are not included in the main figure owing to substantial differences in event rates across donor types and protocols; these are provided in the Supporting Information (Figures S3 and S4) for completeness.

To assess the effect of factors on timing, Cox proportional hazards regression was used (Table 3); hazard ratios (HR) > 1 indicate earlier occurrence (shorter time to event). In the timing models, age ≥ 30 years was associated with earlier tooth loss (HR = 6.56, *p* = 0.007), and male gender with earlier loss of success (HR = 2.42, *p* = 0.026).

**TABLE 3:** Cox proportional hazards regression for complications, loss of success and tooth loss.

Predictor	*N* (group sizes)	Impact on timing (Cox HR; HR > 1 = earlier occurrence)																	
		Inflammatory resorption (EIR/IIR)			Replacement resorption (RR)			Cervical resorption (ECR)			Apical pathology (AP)			Success loss			Tooth loss		
		HR	95% CI	*p*	HR	95% CI	*p*	HR	95% CI	*p*	HR	95% CI	*p*	HR	95% CI	*p*	HR	95% CI	*p*
**Patient‐related factors**																			
Age: ≥ 30 years (vs. < 18 years)	22 vs. 88	2.79	0.97–8.03	0.057	0.32	0.05–2.27	0.253	—	—	0.984	**4.43**	1.39–18.35	**0.014**	1.73	0.63–4.72	0.285	**6.56**	1.69–25.53	**0.007**
Age: 18–29 years (vs. < 18 years)	24 vs. 88	0.79	0.18–3.54	0.758	0.70	0.15–3.24	0.647	0.55	0.07–4.39	0.575	1.79	0.47–6.80	0.392	1.25	0.46–3.36	0.663	2.04	0.39–10.58	0.396
Gender: male (vs. female)	61 vs. 73	**3.50**	1.25–9.75	**0.017**	1.77	0.58–5.42	0.321	3.02	0.76–12.03	0.118	1.65	0.57–4.77	0.358	**2.42**	1.11–5.25	**0.026**	2.05	0.60–7.06	0.254
**Tooth‐related factors**																			
Root development: early 1–3 (vs. optimal 4–5)	11 vs. 71	0.59	0.08–4.68	0.619	1.60	0.33–7.73	0.558	—	—	0.990	—	—	0.981	1.28	0.36–4.57	0.707	—	—	0.986
Root development: late 6–7 (vs. optimal 4–5)	52 vs. 71	1.43	0.57–3.60	0.451	0.81	0.24–2.76	0.733	0.56	0.15–2.18	0.404	1.81	0.66–5.02	0.252	1.61	0.74–3.48	0.230	2.65	0.77–9.06	0.121
Donor tooth type: molar (vs. premolar)	48 vs. 74	0.97	0.37–2.56	0.950	0.87	0.23–3.23	0.832	1.20	0.30–4.85	0.796	1.79	0.56–5.70	0.328	1.05	0.45–2.45	0.905	2.01	0.53–7.63	0.303
Donor tooth type: anterior (canines, incisors, supernumerary from premaxilla; vs. premolar)	12 vs. 74	—	—	0.979	0.88	0.11–7.03	0.901	1.51	0.17–13.09	0.710	**4.44**	1.09–18.14	**0.038**	1.58	0.46–5.46	0.470	3.56	0.67–19.03	0.137
**Treatment‐related factors**																			
Treatment protocol: 2010–present (vs. 2000–2010)	51 vs. 83	1.29	0.52–3.21	0.586	0.70	0.22–2.28	0.558	0.44	0.09–2.09	0.303	0.87	0.30–2.56	0.802	1.04	0.45–2.21	0.921	0.66	0.18–2.49	0.538
Indication: impaction (vs. agenesis/trauma)	10 vs. 72	—	—	0.983	1.94	0.22–17.08	0.550	2.45	0.27–22.60	0.430	2.66	0.30–23.64	0.381	0.98	0.13–7.62	0.984	—	—	0.989
Indication: restorative (vs. agenesis/trauma)	52 vs. 72	1.64	0.66–4.06	0.288	1.05	0.31–3.54	0.939	1.00	0.25–4.08	0.998	**3.61**	1.20–10.88	**0.022**	1.25	0.58–2.70	0.567	1.68	0.50–5.63	0.398
Timing: conventional (vs. immediate)	48 vs. 80	0.83	0.31–2.20	0.701	1.61	0.54–4.79	0.396	1.14	0.32–4.10	0.844	1.09	0.39–3.08	0.865	—	—	—	2.99	0.87–10.21	0.081
Timing: semi‐immediate (vs. immediate)	6 vs. 80	1.19	0.15–9.21	0.869	—	—	0.989	—	—	0.991	—	—	0.984	0.85	0.40–1.80	0.666	—	—	0.986
RCT before transplantation or ≤ 14 days (vs. > 14 days or symptom triggered; mature teeth only)	8 vs. 44	1.05	0.13–8.63	0.962	0.04	0–635,207	0.707	6.07	0.38–97.19	0.203	1.11	0.13–9.25	0.925	1.79	0.38–8.42	0.460	1.11	0.11–9.25	0.923
Antibiotics: amoksicilin 1 g, single dose before (vs. none)	65 vs. 17	**0.14**	0.04–0.46	**0.001**	0.79	0.16–3.87	0.768	0.80	0.09–7.45	0.842	**0.17**	0.04–0.72	**0.017**	1.08	0.36–3.22	0.888	0.51	0.06–4.39	0.537
Antibiotics: amoksicilin 500 mg/1 g, 7 days (vs. none)	52 vs. 17	**0.34**	0.12–0.98	**0.045**	1.02	0.18–5.66	0.982	1.99	0.22–18.10	0.542	0.50	0.14–1.83	0.296	0.53	0.22–1.26	0.148	0.52	0.15–1.85	0.314
Splinting method: suture only (vs. wire)	118 vs. 16	0.95	0.22–4.12	0.944	23.59	0.01–78,949	0.445	24.29	—	0.487	0.72	0.16–3	0.670	1.65	0.39–7.01	0.495	1.01	0.13–8.00	0.989
Splinting time: ≤ 4 weeks (vs. > 4 weeks)	7 vs. 127	0.05	0.00–164.6	0.459	0.05	0–929	0.540	0.04	0.00–1530	0.555	—	—	0.483	0.04	0.00–20.22	0.315	0.05	0.00–164.6	0.459
Orthodontic loading: early (< 3 months; vs. none)	44 vs. 42	0.94	0.31–2.80	0.908	1.43	0.26–7.84	0.683	0.35	0.03–3.86	0.391	0.62	0.17–2.34	0.484	1.00	0.37–2.71	0.993	0.39	0.07–2.12	0.272
Orthodontic loading: conventional (≥ 3 months; vs. none)	24 vs. 42	0.40	0.08–1.97	0.257	3.19	0.62–16.52	0.167	2.42	0.44–13.34	0.310	1.61	0.48–5.49	0.444	1.47	0.50–4.27	0.483	0.84	0.18–3.84	0.822
Surgeon's experience: ≥ 20 cases (vs. < 20 cases)	98 vs. 36	0.67	0.23–1.90	0.445	2.16	0.28–17.01	0.464	0.74	0.15–3.69	0.713	1.36	0.30–6.25	0.692	0.67	0.26–1.72	0.408	0.54	0.14–2.14	0.379
Other variables (root resection, grinding during or after surgery, composite bite stops, restoration, etc.)		—	—	> 0.05	—	—	> 0.05	—	—	> 0.05	—	—	> 0.05	—	—	> 0.05	—	—	> 0.05

*Note:* Bold indicates significant results (*p* < 0.05). Reference categories are shown in parentheses. ‘—’ denotes non‐estimable contrasts (sparse data or no events).

Abbreviations: CI, confidence interval; HR, hazard ratio; RCT, root canal treatment.

For inflammatory resorptions (EIR/IIR), male gender was associated with earlier diagnosis (HR = 3.50, *p* = 0.017). Antibiotic prophylaxis was associated with a later diagnosis of infection‐related complications (Table S2; EIR/IIR: HR = 0.22, *p* = 0.002; AP: HR = 0.29, *p* = 0.046). A single preoperative dose of amoxicillin 1 g administered approximately 1 h before surgery corresponded to a lower hazard (EIR/IIR: HR = 0.14, *p* = 0.001; AP: HR = 0.17, *p* = 0.017), indicating delayed occurrence; a 7‐day post‐operative regimen (amoxicillin 500–1000 mg; clindamycin 300–600 mg if penicillin‐allergic) likewise showed a reduced hazard (EIR/IIR: HR = 0.34, *p* = 0.045). For AP, age ≥ 30 years (HR = 5.05, *p* = 0.014) and anterior donor teeth (HR = 4.44, *p* = 0.038) were associated with earlier diagnosis; restorative indication (e.g., caries or previous RCT failure) was also associated with earlier diagnosis (HR = 3.61, *p* = 0.022). No significant predictors were identified for RR or ECR; wide confidence intervals reflect limited statistical power.

Other patient‐, tooth‐ and treatment‐related factors were not significant; sparse‐data variables with unstable estimates (very wide confidence intervals) are grouped in a summary row at the bottom of Table 3 and are not interpreted individually. Supplementary analyses included a binary specification for any antibiotic prophylaxis (yes/no), canine‐only sensitivity analysis (replacing the anterior group), and an orthodontic loading specification (early < 3 months vs. conventional ≥ 3 months); results are reported in Table S2. In the canines‐only analysis, effect estimates were directionally similar to those of the anterior group but were not statistically significant, with wide confidence intervals owing to the small sample size.

## 4 Discussion

This single‐centre retrospective cohort provides one of the first detailed analyses of the timing of complications following tooth transplantation, with a focus on factors associated with earlier diagnosis. Timing was characterised using event‐only 1–KM summaries and Cox proportional hazards models (with HR > 1 interpreted as earlier occurrence), allowing quantification of how soon complications were first detected rather than simply whether they occurred.

Owing to its single‐centre design and comprehensive review of institutional records spanning 2003–2024, follow‐up information was collected across multiple clinical departments—including operative dentistry, prosthodontics, orthodontics, paediatric dentistry and periodontology—covering both preventive check‐ups and continuation of treatment. This approach enabled a more precise reconstruction of follow‐up histories, including the timing and management of post‐operative complications beyond the scheduled follow‐up appointments with the surgical team, thereby providing a more complete picture of post‐transplantation outcomes. The extended observation period also enabled the inclusion of rare late complications and evaluation of long‐term outcomes in teeth with previously treated complications.

The present cohort included a wide spectrum of cases, ranging from young to adult patients and encompassing various donor tooth types, including premolars, molars and anterior teeth—a heterogeneous group of predominantly single‐rooted, single‐canal donors (incisors, canines and one premaxillary supernumerary tooth). In contrast to studies focusing primarily on young and adolescent populations, a different definition of success was applied. In growing patients, maintaining the transplanted tooth until completion of skeletal growth is often considered a successful outcome, with the absence of ankylosis (RR) serving as a key determinant of space preservation and normal alveolar development [33].

In the present study, however, a deliberately strict definition of success was adopted, based on Tsukiboshi's criteria [11] and interpreted as the absence of any need for further intervention or signs of progressive deterioration. Although successfully treated complications formally meet existing criteria for success [10], clinical experience and long‐term observation indicate that such cases may subsequently require retreatment or develop other types of complications, as observed within the present cohort. Consequently, these cases were classified as losses of success.

The impact of this strict success definition is reflected in the comparison of KM plots for survival and success. While survival outcomes across donor tooth types were consistent with those reported in previous literature [14, 34, 35, 36], the comparatively lower success rate reflects the strict definition applied. Cases requiring retreatment or management of a new complication after initial resolution were not classified as successful, as such findings were interpreted as signs of progressive deterioration and potential progression towards failure. It should also be acknowledged that teeth surviving with complications demonstrated follow‐up durations comparable to those without complications. Many such teeth remained functional and asymptomatic for several years, with long‐term survival—often beyond 5 or even 7 years—despite the presence of slowly progressing pathology. However, gradual progression may ultimately lead to failure in the long term.

Beyond survival and success, the timing of major post‐operative complications represents a key finding of the present study. The complications monitored—most of which could be diagnosed radiographically, although clinical evaluation was also required—included AP and various resorptive processes. Resorption develops following damage to the precementum or predentine layer, often resulting from surgical manipulation during transplantation, with subsequent inflammation of adjacent soft tissues permitting clastic cell invasion [24]. Inflammatory resorptions, owing to their association with bacterial infection of necrotic pulp tissue during or after the procedure [24, 37], tend to occur early [5, 38], with more than half diagnosed within the first year post‐surgery in the present cohort. In contrast, AP—associated with secondary infection of necrotic pulp tissue in the absence of structural damage—tended to develop later. RR is a gradual process in which tooth structure is progressively replaced by bone, resulting in ankylosis [24, 26, 39]. In the present cohort, it was typically diagnosed later in the post‐operative period. Early manifestations were often identified by orthodontists, when tooth movement proved impossible, or by the surgical team during post‐operative reviews using Periotest evaluation or radiographic assessment. ECR, being a slow invasive process, also tended to occur late after transplantation. It originates from vascularised soft tissue at the cemento‐enamel junction, where precementum is absent—most likely as a result of surgical manipulation in transplantation cases—allowing tissue ingrowth into the cervical region, which may later undergo osseous replacement [27, 40]. This results in a mixed radiolucent appearance on radiographs, characteristic of the resorptive process [28].

Half of all complications occurred within the first 18 months, underscoring the importance of structured and closely scheduled follow‐up during this period. Most complications, aside from RR [26], are potentially manageable if detected in their early stages. Therefore, an intensified recall protocol within the first 2 years after transplantation should be considered to enable timely diagnosis and intervention.

Inflammatory resorptions can often be successfully treated by conventional endodontic therapy, provided that treatment is initiated promptly [6, 11]. Early intervention is essential, as advanced EIR may progress to RR and heal by ankylosis following treatment [38]—a finding frequently observed within the present cohort. AP can also be managed effectively with endodontic therapy. Given the technical challenges associated with resorptive defects (e.g., irregular apical constriction, canal obliteration in AP cases), these procedures should ideally be performed by an experienced endodontist.

For ECR, early detection is likewise key to achieving a favourable prognosis, as advanced lesions are frequently untreatable [8, 41]. In these cases, clinical examination plays an important role alongside radiographic assessment—preferably supplemented by three‐dimensional imaging—to confirm the diagnosis and determine the lesion's location and extent. By contrast, RR demonstrated an unfavourable outlook [26, 39]. In the present cohort, all treated cases—despite temporary improvement following surgical luxation and orthodontic repositioning—exhibited recurrence or further progression, often in combination with ECR. The only tooth that remained mobile at final follow‐up lacked sufficient observation time to confirm long‐term stability.

Although predictors of whether complications occur have been described, less is known about whether specific predictors influence how early or late complications arise during follow‐up; therefore, this study focused on timing. In time‐to‐event analyses, several patient‐, tooth‐ and treatment‐related factors were associated with earlier occurrence of post‐operative complications. Age ≥ 30 years was linked to an increased hazard of tooth loss (i.e., earlier extraction; Table 3). This finding is consistent with previous studies reporting less favourable outcomes in older patients [10, 14, 42, 43], often attributed to reduced healing capacity, limited availability of suitable donor teeth, greater difficulty in achieving atraumatic extraction, and a more frequent need for technically challenging RCT due to complex or calcified canal anatomy. In this group, AP was also diagnosed earlier than in younger patients.

Male gender was associated with a higher hazard for earlier occurrence of inflammatory resorptions and earlier loss of success (Table 3). The male and female groups were comparable in age and donor tooth distribution (Table 1), suggesting that this association may reflect biological and/or behavioural differences rather than differences in baseline characteristics [44]. From a biological perspective, sex‐hormone–related differences in clastic activity may contribute; lower oestrogen signalling—known to down‐regulate osteoclastogenesis in PDL cells—could contribute to a faster progression of resorptive processes [45]. Behaviourally, missed or delayed follow‐up visits were more common among male patients in this cohort, suggesting lower compliance and a more symptom‐driven approach to attendance. The observations are associative, not causal, and the possible biological and behavioural bases warrant further investigation.

Restorative indications (e.g., caries or previous RCT failure) were associated with a higher hazard of earlier AP (Table 3). This association is plausibly multifactorial: restorative cases tend to involve older patients (more complex canals, calcifications) and a higher bacterial burden at the recipient site—including residual intraradicular or apical infection in the socket and inflamed peri‐socket soft tissues that can harbour biofilm. These conditions may shift AP to earlier diagnosis despite standard protocols [46]. These explanations remain hypothetical and merit additional study.

Anterior donor teeth—a heterogeneous group of predominantly single‐rooted, single‐canal donors (incisors, canines and one premaxillary supernumerary tooth)—showed earlier AP (Table 3). In a canines‐only sensitivity, estimates were directionally similar but not statistically significant owing to the small sample size and wide confidence intervals. Several case characteristics may contribute: a higher proportion of impactions and a greater share of late root development (late:early/optimal ≈2:1; Table 1) [47]. Difficult extractions—due to unfavourable tooth position, sometimes following unsuccessful orthodontic traction—can entail crown microtrauma, potentially facilitating bacterial ingress during the early healing phase; close observation for potential revascularisation and deferral of RCT in Moorrees stage 6 may also have contributed to earlier diagnosis. These interpretations are associative and hypothesis‐generating.

On the other hand, antibiotic prophylaxis was associated with later diagnosis infection‐related events (EIR/IIR, AP). In both specifications—any prophylaxis versus none (Table S2) and models separating a single preoperative amoxicillin 1 g dose from a 7‐day post‐operative regimen (amoxicillin 500–1000 mg; clindamycin 300–600 mg if penicillin‐allergic; Table 3)—a delay in diagnosis was observed, with the single dose strategy appearing sufficient in this cohort. A plausible mechanism is the transient reduction of bacterial load during early healing, when pulp revascularisation is initiated via an open apex [19]; limiting contamination at this stage may delay infection‐related complications. These findings align with prior reports that higher dose amoxicillin (e.g., 3 g) reduces endodontic complications in immature teeth [1, 2].

No significant difference in the timing of infection‐related complications was observed between mature teeth (late Moorrees' stages 6–7) managed with RCT performed before or within 14 days and those treated with latency. As shown in Table S1, early RCT was associated with fewer infection‐related complications, aligning with reports by Barendregt and Louropoulou that RCT performed before or within 14 days can virtually eliminate such complications [1, 2, 19]. However, in our cohort one molar still failed from inflammation despite adherence to this protocol (*n* = 8 in this subgroup; interpret with caution), underscoring that case selection and anatomical complexity remain critical—planning for the feasibility of RCT is as important as protocol timing. Notably, once infection occurred, its timing was similar across RCT strategies (Table 3; Figure S4), indicating that onset is driven primarily by biological factors, with procedural scheduling playing a lesser role.

For clinical practice, the findings of this study underscore the importance of a well‐structured post‐operative follow‐up protocol led by the surgical team, particularly within the first 2 years post‐transplantation. Half of all complications were diagnosed within 18 months; later onset entities (ECR, AP and RR) had median timings of approximately 18–21 months, indicating that most events arise during the first 2 years. Consequently, a condensed recall schedule—such as maintaining 3‐month intervals throughout the first 2 years—is advisable to facilitate timely diagnosis and management. Equally important is the need to educate other specialists and referring dentists involved in the patient's ongoing care to ensure effective communication and coordinated monitoring. Patients should be informed about the importance of continued long‐term follow‐up, as certain complications, including ECR, may still develop several years after transplantation. Furthermore, the possibility of complication recurrence or transformation into a different type should be considered during follow‐up. Careful case selection—including preoperative evaluation of the feasibility of planned RCT in mature teeth—together with atraumatic surgical handling, is a key determinant of success. Groups showing earlier complication onset—such as older patient age or anterior donors (predominantly canines)—likely reflect greater anatomical and procedural difficulty and therefore warrant tailored planning.

### 4.1 Limitations

Owing to its retrospective design, this study has several limitations. Estimates beyond 7 years of follow‐up should be interpreted with caution, as the number of cases decreased substantially due to censoring (i.e., loss to follow‐up). The reliance on radiological diagnosis should also be acknowledged; although clinical records were reviewed, detailed periodontal charts were unavailable for older cases due to the clinic's earlier use of hybrid documentation—paper charts (now discarded) complemented by brief electronic records. Furthermore, the wide spectrum of indications and donor tooth types may have contributed to variability in outcomes. The comparatively low success rate may reflect the deliberately strict definition of success adopted, along with the conservative, worst‐case classification of teeth still undergoing complication treatment at the last recorded follow‐up, which were not counted as successful unless full resolution was documented. Finally, owing to the limited number of events, the influence of perioperative factors on the timing of complication diagnosis could not be reliably estimated. Prospective studies with larger samples could further clarify their role in time‐to‐complication trends.

## 5 Conclusion

Post‐operative complications present a significant challenge in tooth autotransplantation. Early detection influences outcomes, as many complications are manageable and compatible with long‐term success. Half of all complications were diagnosed within 18 months (median time to first diagnosis: 11.7 months for EIR/IIR; 19.6 months for AP; 18.0 months for ECR; 21.4 months for RR). Accordingly, a condensed recall schedule—maintaining three‐monthly intervals throughout the first 2 years—is advisable to facilitate timely diagnosis and management, with long‐term monitoring thereafter for late changes, notably ECR and RR.

## Author Contributions

**Juraj Marton:** conceptualisation, methodology, validation, formal analysis, investigation, resources, data curation, writing – original draft preparation, writing – review and editing, visualisation. **Radovan Žižka:** validation, formal analysis, writing – review and editing. **Linda Kučerová:** investigation, writing – review and editing. **Přemysl Krejčí:** formal analysis, writing – review and editing. **Martin Starosta:** writing – review and editing. **Zdeněk Pokorný:** supervision. All authors have read and agreed to the published version of the manuscript.

## Funding

The authors have nothing to report.

## Ethics Statement

The study and publication were approved by the Ethics Committee of the University Hospital and the Faculty of Medicine, Palacký University in Olomouc under reference number 105/25. As this was a retrospective observational study involving anonymised data, the requirement for individual patient consent was waived by the committee.

## Conflicts of Interest

The authors declare no conflicts of interest.

## Supporting information


**Appendix S1:** edt70038‐sup‐0001‐AppendixS1.docx.

## Data Availability

The data that support the findings of this study are available from the corresponding author upon reasonable request.
