# Supplementary material for: Prevalence of Post‐Operative Complications in Autotransplanted Teeth: A Long‐Term Retrospective Cohort
Source: Dent Traumatol. 2025 Dec 2;42(4):461–75. doi: 10.1111/edt.70038 (PMC13356525; doi:10.1111/edt.70038)
Supplement: Supplementary file 1 — Appendix S1: edt70038‐sup‐0001‐AppendixS1.docx. [file EDT-42-461-s001.docx]

**Supplementary Tables:**

| **Status** |  | **Early (1–3; n=11)** | **Optimal (4–5; n=71)** | **Late (6–7) RCT >14 days or symptom-triggered (n=44)** | **Late (7) RCT before or ≤14 days (n=8)** | **All (n=134)** | **Co-occurring / subsequent events** |
| --- | --- | --- | --- | --- | --- | --- | --- |
| **Surviving** |  | 11 (100%) | 67 (94.4%) | 37 (84.1%) | 7 (87.5%) | **122 (91.0%)** |  |
| Without complication |  | 8 (72.7%) | 51 (71.8%) | 25 (56.8%) | 6 (75.0%) | **92 (68.7%)** |  |
| With complication |  | 3 (27.3%) | 16 (22.5%) | 12 (27.2%) | 1 (12.5%) | **32 (23.9%)** |  |
| Successfully treated | EIR |  | 6 (7.0%) | 3 (6.8%) |  | 9 (6.7%) | Optimal: 2 subsequently developed RR; Late, RCT delayed: 1 subsequently failed due to RR |
|  | IIR |  | 1 (1.4%) |  |  | 1 (0.7%) |  |
|  | AP |  | 5 (7.0%,) | 3 (6.8%) |  | 8 (5.9%) | Optimal: 2 also had EIR (treated); Late, RCT delayed: 1 subsequently developed RR and 1 subsequently failed due to EIR |
|  | RR |  | 1 (1.4%) |  |  | 1 (0.7%) |  |
|  | ECR |  | 2 (2.8 %) | 1 (2.3%) |  | 3 (2.2%) | Optimal: 1 subseqently failed due to AP+IIR |
| Progressing / under treatment | EIR |  |  | 2 (4.5%) |  | 2 (1.5%) |  |
|  | IIR | 1 (9.1%) |  | 1 (2.3%) |  | 2 (1.5%) |  |
|  | AP |  | 1 (1.4%) | 3 (6.8%) |  | 4 (3.0%) | Late, RCT delayed: 1 also had EIR and 1 also IIR |
|  | ECR |  | 3 (4.2%) | 1 (2.3%) | 1 (12.5%) | 5 (3.7%) |  |
|  | RR | 2 (18.2%) | 4 (5.6%) | 3 (6.8%) |  | 9 (6.7%) | Optimal: 1 also had ECR |
| **Tooth loss (extraction)** |  |  | 4 (5.6%) | 6 (13.6%) | 2 (25.0%) | **12 (9.0%)** |  |
| (progressing complications present at the day of extraction) | EIR |  |  | 3 (6.8%) | 1 (12.5%) | 4 (3.0%) |  |
|  | IIR |  | 2 (2.8%) |  |  | 2 (1.5%) |  |
|  | AP |  | 1 (1.4%) | 1 (2.3%) | 1 (12.5%;) | 3 (2.2%) | Optimal: 1 also had IIR; Late, RCT delayed: 1 also had AL; Late, RCT early: one also had EIR |
|  | RR |  | 2 (2.8%) | 1 (2.3%) |  | 3 (2.2%) | Optimal: 2 also had ECR |
|  | ECR |  | 2 (2.8%) |  |  | 2 (1.5%) |  |
|  | AL |  |  | 3 (6.8%) |  | 3 (2.2%) |  |
| **Teeth with ≥2 complications** |  |  | 8 (11.3%) | 6 (13.6%) | 1 (12.5%) | 15 (11.2%) |  |

**Supplementary Table S1.** Status of autotransplanted teeth at last recall: overview of complications by Moorrees’ stage and timing of root canal treatment (RCT).

Abbreviations: EIR, external inflammatory resorption; IIR, internal inflammatory resorption; AP, apical pathology; RR, replacement resorption; ECR, external cervical resorption; AL, periodontal attachment loss.
Note: percentages are calculated column-wise.

| **Predictor** | **N (group sizes)** | **Impact on timing (Cox HR; HR>1 = earlier occurrence):** | | | | | | | | | | | | | | | | | |
| --- | --- | --- | --- | --- | --- | --- | --- | --- | --- | --- | --- | --- | --- | --- | --- | --- | --- | --- | --- |
|  |  | **Inflammatory Resorption (EIR/IIR)** | | | **Replacement Resorption (RR)** | | | **Cervical Resorption (ECR)** | | | **Apical pathology (AP)** | | | **Success loss** | | | **Tooth loss** | | |
|  |  | **HR** | **95% CI** | **p-value** | **HR** | **95% CI** | **p-value** | **HR** | **95% CI** | **p-value** | **HR** | **95% CI** | **p-value** | **HR** | **95% CI** | **p-value** | **HR** | **95% CI** | **p-value** |
| **Sensitivity analyses** |  |  |  |  |  |  |  |  |  |  |  |  |  |  |  |  |  |  |  |
| Orthodontic loading: early (<3 months; vs late ≥3 months) | 44 vs 24 | 2.47 | 0.51–11.90 | 0.262 | 0.46 | 0.12–1.74 | 0.256 | 0.15 | 0.02–1.13 | 0.087 | 0.33 | 0.08–1.31 | 0.114 | 0.61 | 0.22–1.71 | 0.349 | 0.47 | 0.08–2.84 | 0.407 |
| Donor tooth type: canine (vs premolar) | 7 vs 74 | 0.0 | – | 0.984 | 1.58 | 0.20–12.73 | 0.668 | 0.0 | – | 0.990 | 2.61 | 0.31–22.14 | 0.379 | 0.0 | – | 0.984 | 0.0 | – | 0.987 |
| Donor tooth type: molar (vs premolar) | 48 vs 74 | 0.97 | 0.37–2.56 | 0.950 | 0.87 | 0.23–3.23 | 0.833 | 1.183 | 0.29–4.79 | 0.814 | 1.79 | 0.56–5.70 | 0.328 | 1.06 | 0.46–2.47 | 0.889 | 1.97 | 0.52–7.48 | 0.320 |
| Moorrees+RCT timing: early (1–3; vs optimal) | 11 vs 71 | 0.59 | 0.75–4.68 | 0.619 | 1.60 | 0.33–7.73 | 0.558 | 0.0 | – | 0.985 | 0.0 | – | 0.981 | 1.27 | 0.36–5.56 | 0.711 | 0.0 | – | 0.986 |
| Moorrees+RCT timing: late (6–7), RCT >14d (vs optimal) | 44 vs 71 | 1.43 | 0.55–3.72 | 0.458 | 0.91 | 0.27–3.11 | 0.881 | 0.41 | 0.09–1.98 | 0.267 | 1.79 | 0.63–5.11 | 0.277 | 1.51 | 0.68–3.38 | 0.312 | 2.59 | 0.73–9.20 | 2.594 |
| Moorrees+RCT timing: late (7), RCT ≤14d (vs optimal) | 8 vs 71 | 1.37 | 0.17–10.92 | 0.764 | 0.0 | – | 0.989 | 2.11 | 0.25–17.75 | 0.493 | 2.00 | 0.24–16.69 | 0.520 | 2.50 | 0.55–11.33 | 0.235 | 3.02 | 0.33–27.39 | 3.016 |
| Any antibiotic prophylaxis (vs none) | 117 vs 17 | **0.22** | 0.08–0.56 | **0.002** | 0.86 | 0.19–3.94 | 0.848 | 1.18 | 0.15–9.59 | 0.878 | **0.29** | 0.09–0.98 | **0.046** | 0.63 | 0.24–4.45 | 0.951 | 1.37 | 0.17–10.81 | 0.767 |
| Recipient tooth present (vs not present = semiimediate + conventional) | 54 vs 80 | 1.16 | 0.46–2.95 | 0.756 | 0.71 | 0.24–2.10 | 0.531 | 0.98 | 0.27–3.51 | 0.971 | 1.00 | 0.35–2.80 | 0.994 | 1.12 | 0.59–2.10 | 0.736 | 0.37 | 0.11–1.27 | 0.114 |

**Supplementary Table S2.** Cox proportional hazards regression – sensitivity analyses.

Note: **Bold** indicates significant results (p < 0.05). Reference categories are shown in parentheses. “–“ denotes non-estimable contrasts (sparse data or no events).
Abbreviations: HR, hazard ratio; CI, confidence interval; RCT, root canal treatment.

**Supplementary Figures:**


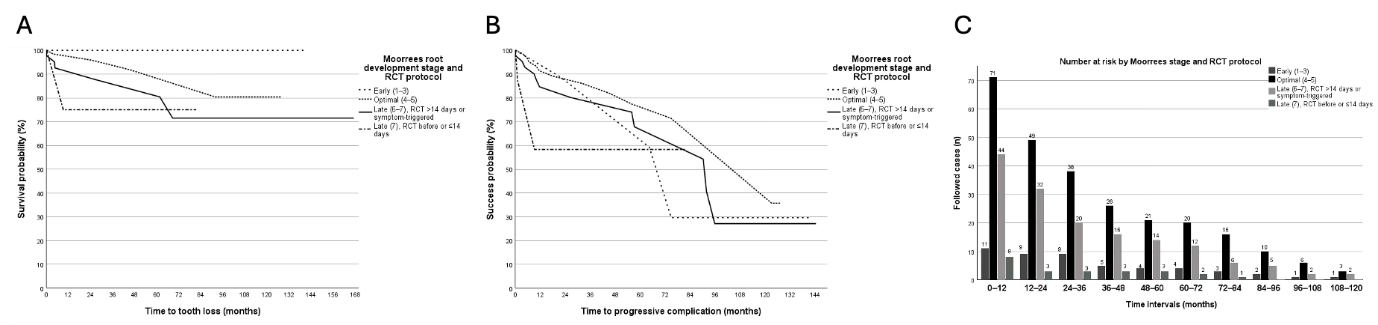


**Supplementary Figure S1.** Kaplan–Meier survival and success curves stratified by Moorrees’ stage and timing of root canal treatment (RCT).

(a) Survival; (b) Success; (c) Numbers at risk (12-month intervals). No between-group differences were observed (log–rank: survival p = 0.255; success p = 0.446).


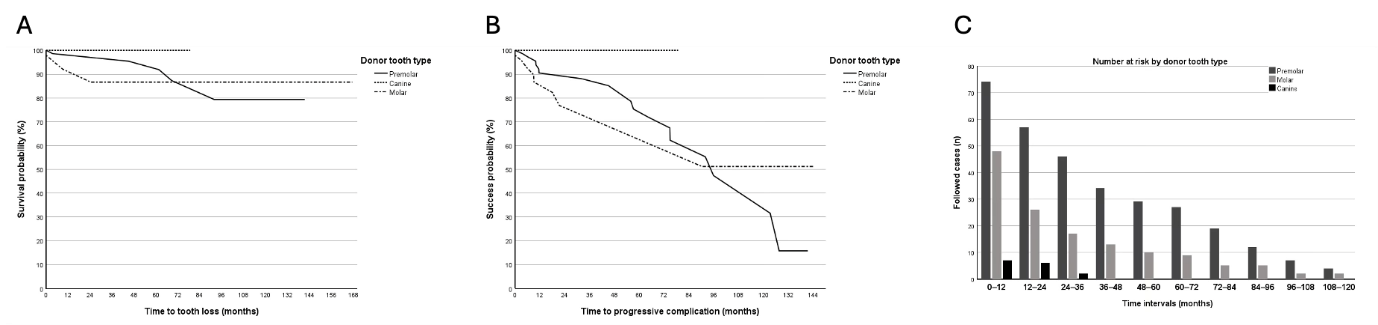


**Supplementary Figure S2.** Sensitivity analysis: Kaplan–Meier curves stratified by donor tooth type (canines only).

(a) Survival; (b) Success; (c) Numbers at risk (12-month intervals). Log–rank test were not reported due to small sample size or non-estimable comparisons.


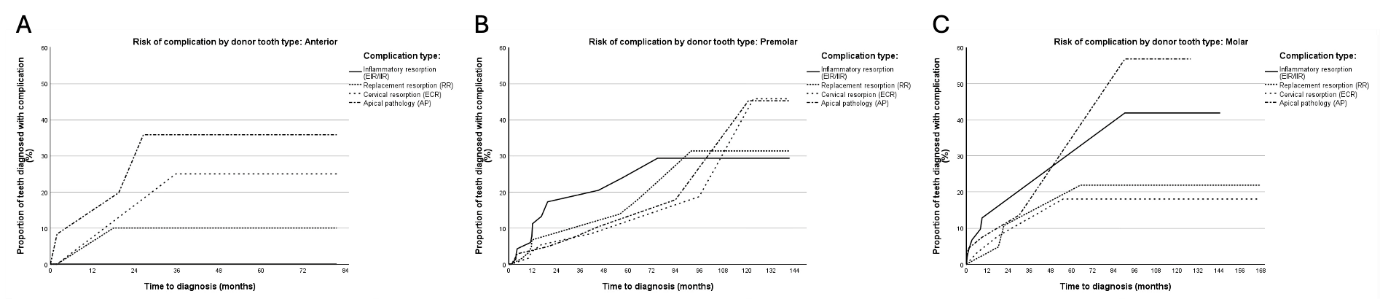


**Supplementary Figure S3.** Full cohort cumulative incidence (1–Kaplan–Meier) of postoperative complications, stratified by donor tooth type.

(a) Anterior (incisors, canines, one premaxillary supernumerary); (b) Premolars; (c) Molars. Separate curves are shown for each complication type; external and internal inflammatory resorption are combined owing to small numbers.


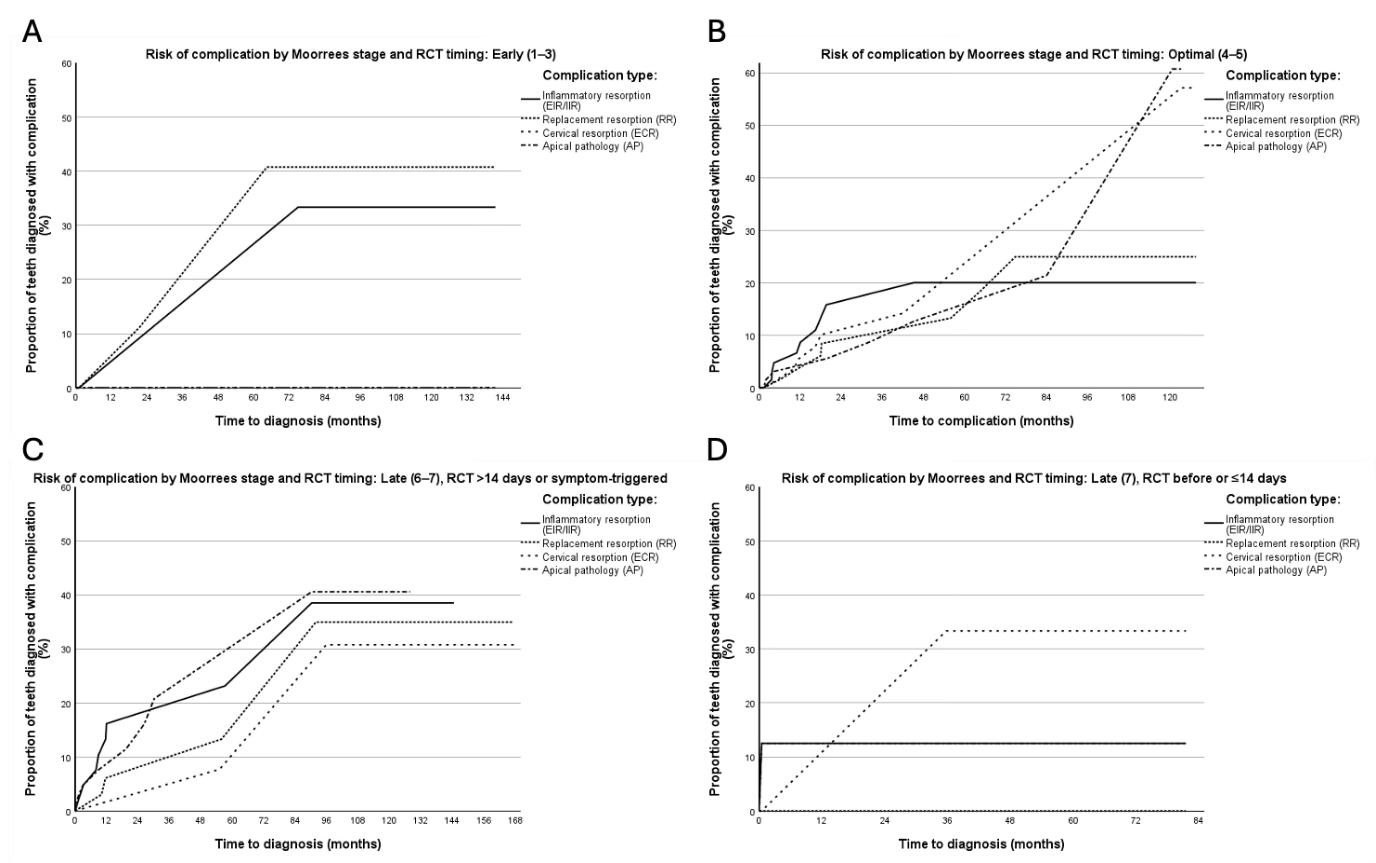


**Supplementary Figure S4.** Full cohort cumulative incidence (1–Kaplan–Meier) of postoperative complications, stratified by Moorrees’ stage and root canal treatment (RCT) timing.

(a) Early (1–3); (b) Optimal (4–5); (c) Late (6–7), RCT >14 days or symptom-triggered; (d) Late (7), RCT ≤ 14 days. Separate curves are shown for each complication type; external and internal inflammatory resorption are combined owing to small numbers.
